# Navigating Heart Transplantation in a Highly Sensitized Patient with Takayasu Arteritis: The Role of Perioperative Desensitization

**DOI:** 10.3390/jcm15218383

**Published:** 2026-10-28

**Authors:** Michał Wilk, Martyna Kowalczyk, Emilia Kiełczyńska, Magdalena Cielecka, Michał Zakliczyński

**Affiliations:** 1 Institute of Heart Diseases, Wroclaw Medical University, 50-556 Wrocław, Poland; 2 Student Scientific Organization, Institute of Heart Diseases, Wroclaw Medical University, 50-556 Wrocław, Poland; 3 Clinic of Cardiac Transplantation and Mechanical Circulatory Support, Wroclaw Medical University, 50-556 Wrocław, Poland

**Keywords:** Takayasu arteritis, heart transplantation, HLA sensitization, perioperative desensitization

## Abstract

**Introduction:** Takayasu arteritis (TA) is a rare large-vessel vasculitis that in selected patients can lead to severe cardiovascular complications, including advanced heart failure (HF), requiring heart transplantation (HTx). Marked pre-transplant high levels of anti-HLA antibodies and notable sensitization substantially complicate donor selection and may prolong time spent on transplant waitlists. In highly sensitized recipients, HTx may therefore require individualized perioperative desensitization strategies to reduce the risk of antibody-mediated graft injury. **Case Summary:** We present the case of a 46-year-old woman with TA and advanced HF who underwent HTx after individualized perioperative desensitization in the setting of significant pre-transplant sensitization (PRA > 60%). Her postoperative course required intensive clinical and immunological monitoring and treatment. The early PT course was complicated by severe clinical deterioration, including a life-threatening episode of asystole that occurred in temporal association with peak anti-HLA class I and II antibody levels. **Discussion:** The case highlights the complexity of alloimmune management in a patient with TA and marked pre-transplant sensitization. Also, the temporal relationship between peak antibody levels and clinical instability suggests the need for close immune surveillance, tailored desensitization and individualized immunosuppressive treatment strategies in this high-risk group. **Conclusions:** This case describes the clinical course of a highly sensitized patient with TA who underwent HTx following an individualized perioperative desensitization strategy based on a modified Hannover protocol. Although transplantation was followed by substantial postoperative complications, the patient remained clinically stable during follow-up. The efficacy of the modified desensitization strategy cannot be established from a single case.

## 1. Introduction

Takayasu arteritis (TA) is a large-vessel vasculitis that primarily affects the aorta and its major branches [1,2]. The disease most frequently affects young females and is characterized by the formation of inflammatory granulomas within the intima and media of the arterial walls, which can result both in stenotic lesions and aneurysms [3,4,5]. Accordingly, the main complications include arterial hypertension and aortic regurgitation (AR). Although cardiac involvement is less common than vascular manifestations, severe cardiovascular complications may ultimately lead to advanced HF [6]. In selected patients with advanced disease, individualized therapeutic strategies may be required, and, in rare cases, HTx may become a treatment option [7,8].

TA is also associated with complex immune dysregulation, involving both cellular and humoral activity [9,10]. Chronic inflammatory response, where interleukin-6 plays a significant role, may contribute to increased immune reactivity in this group of patients [11,12,13,14]. However, pre-transplant anti-human leukocyte antigen (anti-HLA) alloimmunization generally requires prior exposure to alloantigens, which may result from situations such as pregnancy, blood product transfusion, or previous transplants [15]. Therefore, the presence of an autoimmune disease such as TA may represent an additional immunological challenge in highly sensitized transplant candidates; however, a simple and direct relationship between TA and anti-HLA sensitization cannot be assumed.

**Figure 1:**
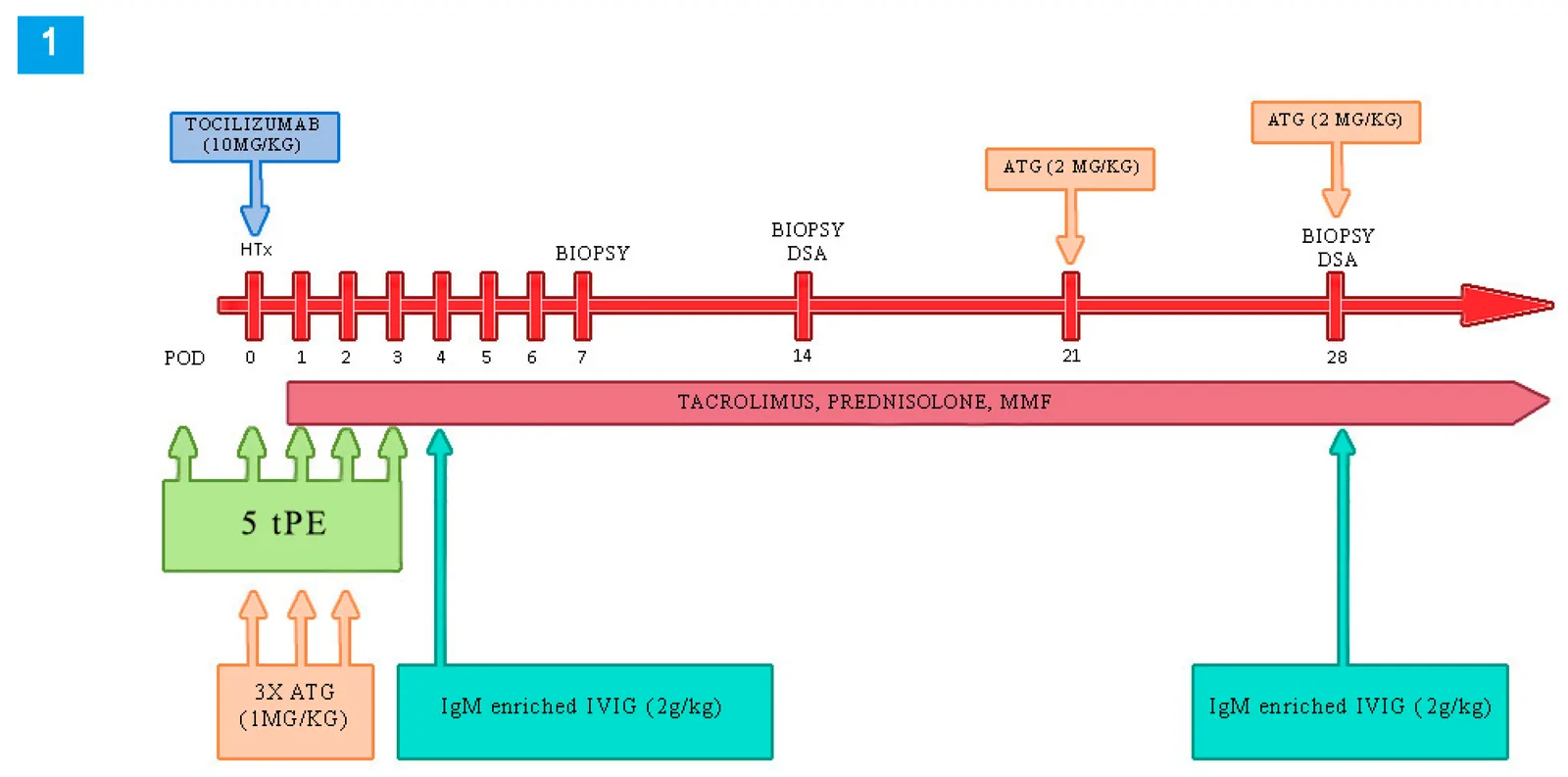
Modified perioperative desensitization protocol based on the Hannover protocol. The figure illustrates the initial treatment plan and does not fully reflect subsequent modifications made in response to postoperative complications.

Preformed anti-HLA antibodies can complicate donor selection in HTx candidates by increasing the risk of immunological incompatibility and limiting the pool of suitable donors [16]. Highly sensitized patients may therefore experience prolonged time on the transplant waitlist, but also an increased risk of complications related to donor incompatibility [17]. In selected patients with previously developed donor-specific antibodies and positive crossmatch, HTx may be considered when the expected benefit of transplantation outweighs the immunological risk. In such a situation, a perioperative desensitization strategy is implemented in order to reduce the risk associated with transplantation [18]. The perioperative desensitization protocol described by Sommer et al. represents one of the approaches reported in the literature for immunological management in highly sensitized HTx candidates [18]. In this manuscript, we refer to this strategy as the “Hannover Protocol”. Accordingly, we present the case of a highly sensitized patient with TA and advanced HF who underwent HTx following an individualized perioperative desensitization strategy based on a modified Hannover protocol (Figure 1).

## 2. Case Presentation

A white 46-year-old Polish female with a history of TA and HF was admitted for routine evaluation due to progressive HF and underlying vascular defects. The available medical records confirmed a history of TA; however, detailed documentation regarding the initial diagnostic work-up, disease activity before transplantation, previous TA-specific treatment, and the extent of residual vascular involvement was unavailable for this retrospective report.

She reported progressive exertional dyspnea over the preceding two months, occurring during minimal activity, such as climbing a few stairs, as well as pressure-like chest pain. The patient’s surgical history included multiple procedures, beginning with supracoronary aortic replacement, followed by reoperation with implantation of a mechanical aortic valve prosthesis (On-X 23 ACE) and a supracoronary vascular graft (Jotec 38) due to infective endocarditis in 2016. In 2018, she underwent surgical closure of a defect in the ascending aortic wall and closure of the pseudoaneurysm sac, reflecting the complexity of her underlying aortic pathology and the burden of previous cardiovascular surgery. Compared with the echocardiographic assessment performed 7 months earlier, left ventricular ejection fraction (EF) had decreased from 62% to 42%.

**Figure 2:**
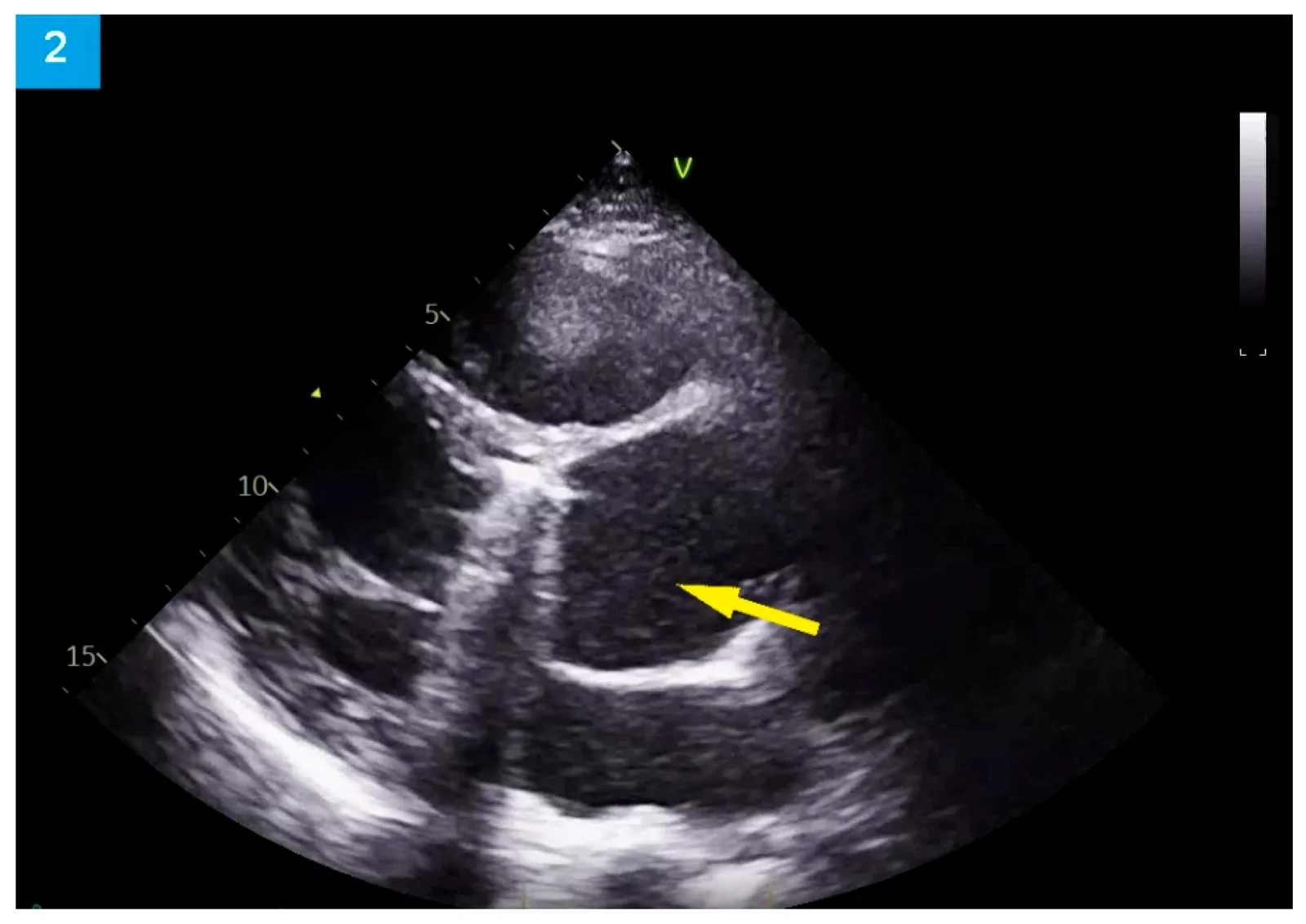
Aortic root aneurysm measuring 58 mm.

**Figure 3:**
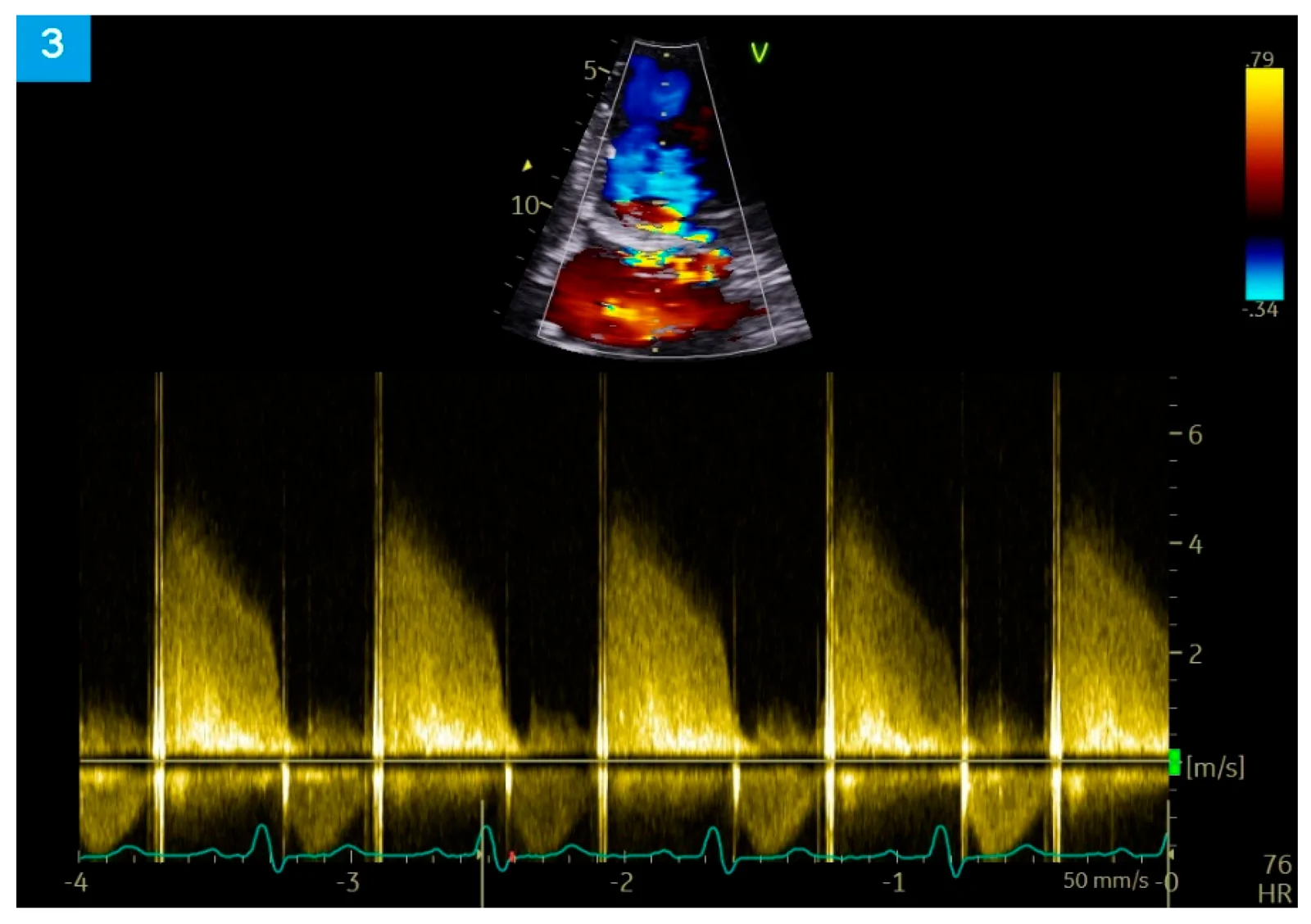
Doppler echocardiography demonstrating aortic regurgitation.

**Figure 4:**
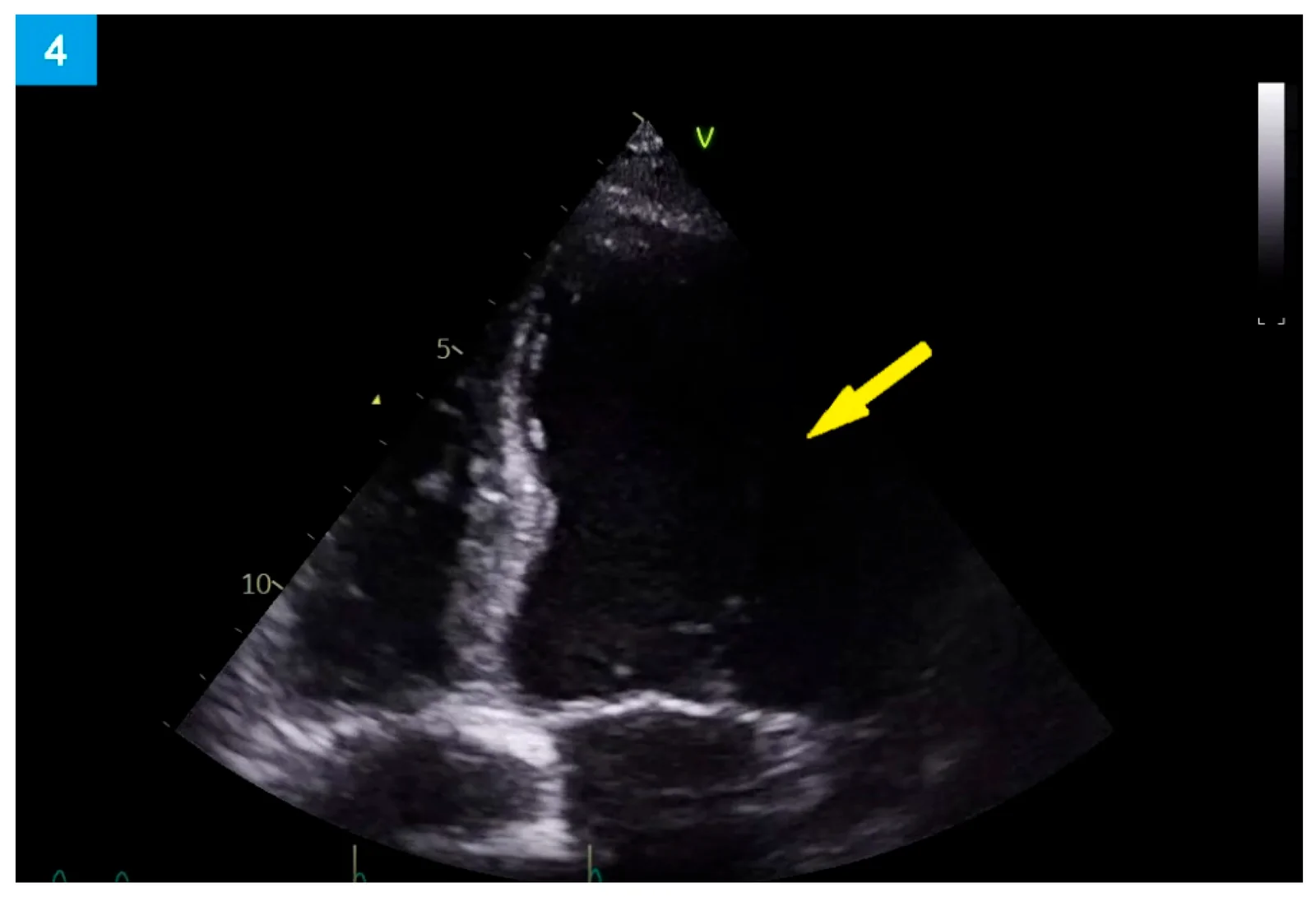
Left ventricular dilation secondary to aortic regurgitation.

**Figure 5:**
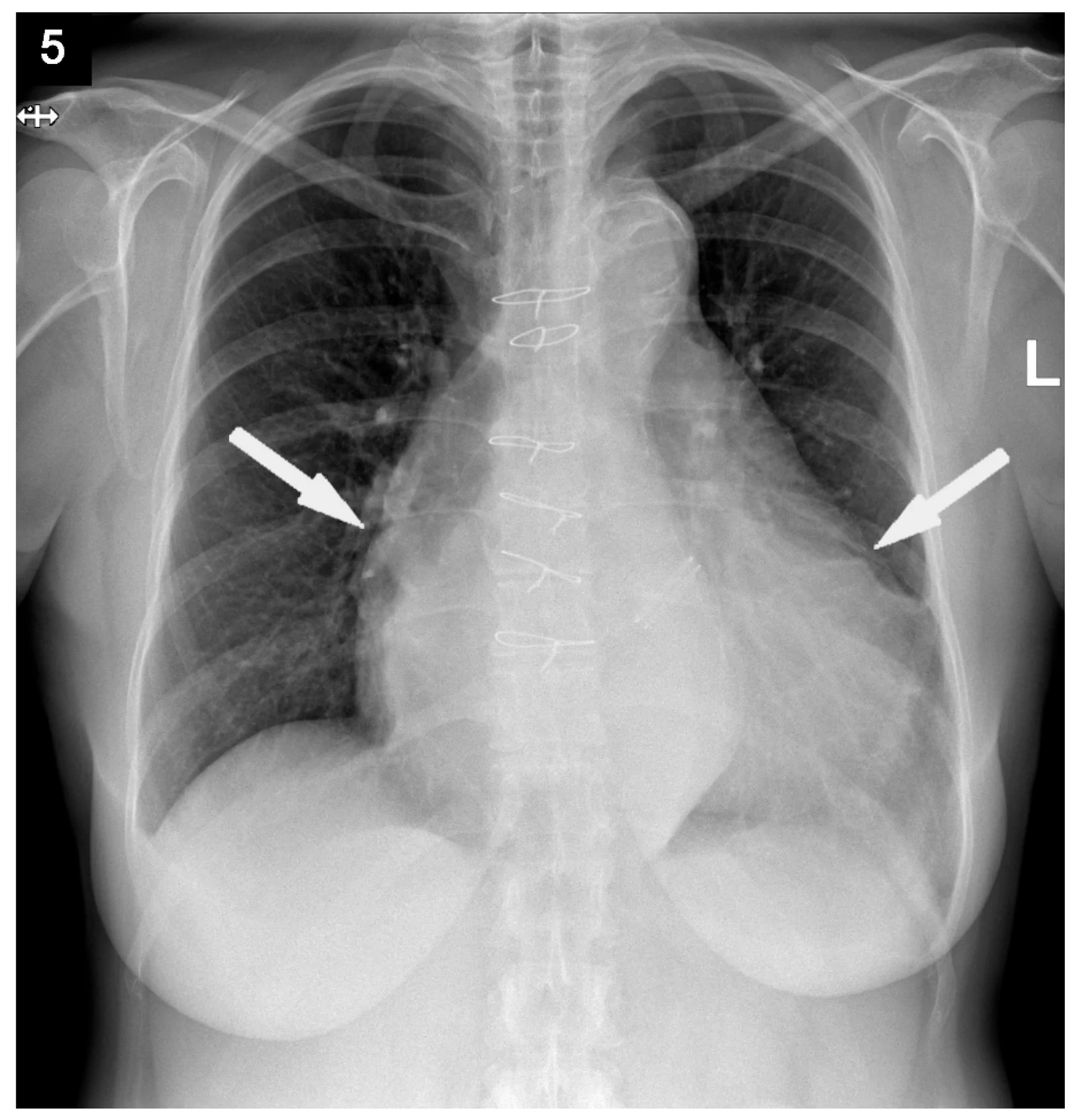
Chest X-ray showing an enlarged heart size prior to transplantation.

On admission, the patient presented with dyspnea (NYHA III), chest pain, hemolytic anemia, aortic paravalvular leak, and peripheral edema. Transthoracic echocardiography (TTE) revealed an EF of 42%, severe AR, moderate mitral regurgitation and severe secondary tricuspid regurgitation. During the examination, a 58 mm aortic root aneurysm was also visualized (Figure 2). Continuous-wave Doppler across the aortic valve showed a dense regurgitant jet with a steep deceleration slope, consistent with severe AR (Figure 3). Additionally, TTE demonstrated left ventricular enlargement with dyssynchronous contraction, grade II diastolic dysfunction, right ventricular enlargement with preserved systolic function, and biatrial enlargement. Left ventricular enlargement was associated with eccentric hypertrophy and increased end-diastolic volume (Figure 4). Additionally, chest X-ray revealed an enlarged cardiac silhouette, along with a fibroinflammatory conglomerate in the anterior mediastinum, which significantly altered the cardiac contour (Figure 5).

Right heart catheterization showed pulmonary artery pressures of 30/15 mmHg (mean 20 mmHg) and a pulmonary vascular resistance of 0.86 Wood units. The six-minute walk distance was 241 m, and the NT-proBNP concentration was 8202 pg/mL. Given the history of multiple previous cardiovascular surgeries, further surgical reconstruction was considered to carry a substantial risk of procedural failure. Therefore, in the context of progressive HF and complex valvular and aortic disease, HTx was considered the preferred treatment strategy.

The patient was highly sensitized, which substantially complicated donor selection. Several potential donors had previously been excluded because of positive complement-dependent cytotoxicity crossmatches (CDC crossmatches). The crossmatch result for the donor ultimately selected for transplantation was not available for this retrospective analysis. However, hospital records confirm that the patient was repeatedly deemed unsuitable for HTx. Nevertheless, given the progressive HF and complex valvular and aortic disease, she was ultimately accepted for Htx candidacy, and her transplant listing status was subsequently upgraded to urgent.

Given the patient’s marked pre-transplant sensitization, anti-HLA class I and II antibodies were regularly monitored. Panel reactive antibodies (PRAs) remained above 60%, precluding identification of a suitable immunologically compatible donor. Anti-HLA antibodies were assessed using a semiquantitative multiplex solid-phase assay (Luminex FLEXMAP 3D, One Lambda, West Hills, CA, USA), which detected IgG antibodies against HLA class I and class II antigens, as well as MICA antigens. Anti-HLA class I and class II antibody results remained positive throughout the monitoring period. The maximum MFI values were 19,309 for HLA class I and 9672 for HLA class II, with corresponding maximum NBG ratios of 386.8 and 193.9, respectively. In contrast, anti-MICA antibody results were initially negative and eventually reached a borderline status.

Therefore, an alternative strategy was pursued. That is why, on 11 March 2023—the day of the transplant—intensive perioperative desensitization was initiated using plasma exchange, anti-thymocyte globulin, intravenous immunoglobulin, and tocilizumab, constituting our modification of the Hannover protocol (Figure 1) [18]. However, the initial desensitization protocol was modified in the context of the patient’s complicated postoperative course and in accordance with the institutional treatment protocol. Post-transplant hemodynamic instability and respiratory failure requiring mechanical ventilation with high inspired oxygen concentrations and high positive end-expiratory pressure, together with worsening renal function requiring loop diuretic therapy, contributed to the decision not to pursue additional plasma exchange sessions beyond the initial desensitization protocol, which were considered after the cardiac arrest. Rituximab was not administered.

Surveillance endomyocardial biopsies (EMBs) were performed according to the institutional post-transplantation (PT) protocol. During the first month after transplantation, four biopsies were scheduled at weekly intervals, followed by two biopsies during the second month at two-week intervals. One biopsy was scheduled during each of the third and fourth months, with subsequent biopsies performed every two months, resulting in a total of 12 surveillance biopsies planned during the first year after transplantation. In this patient, the routine surveillance schedule had to be temporarily modified during the initial PT period because of her clinical course. Nevertheless, closer histopathological monitoring was continued throughout follow-up, and a total of 17 EMBs were performed.

Acute cellular rejection (ACR) was graded according to the original ISHLT classification used at the time of diagnosis. The reported grades (1A, 1B, and 3A) are retained as documented in the medical records. Under the revised ISHLT grading system, the historical grades 1A and 1B correspond to grade 1R, whereas grade 3A corresponds to grade 2R.

**Figure 6:**
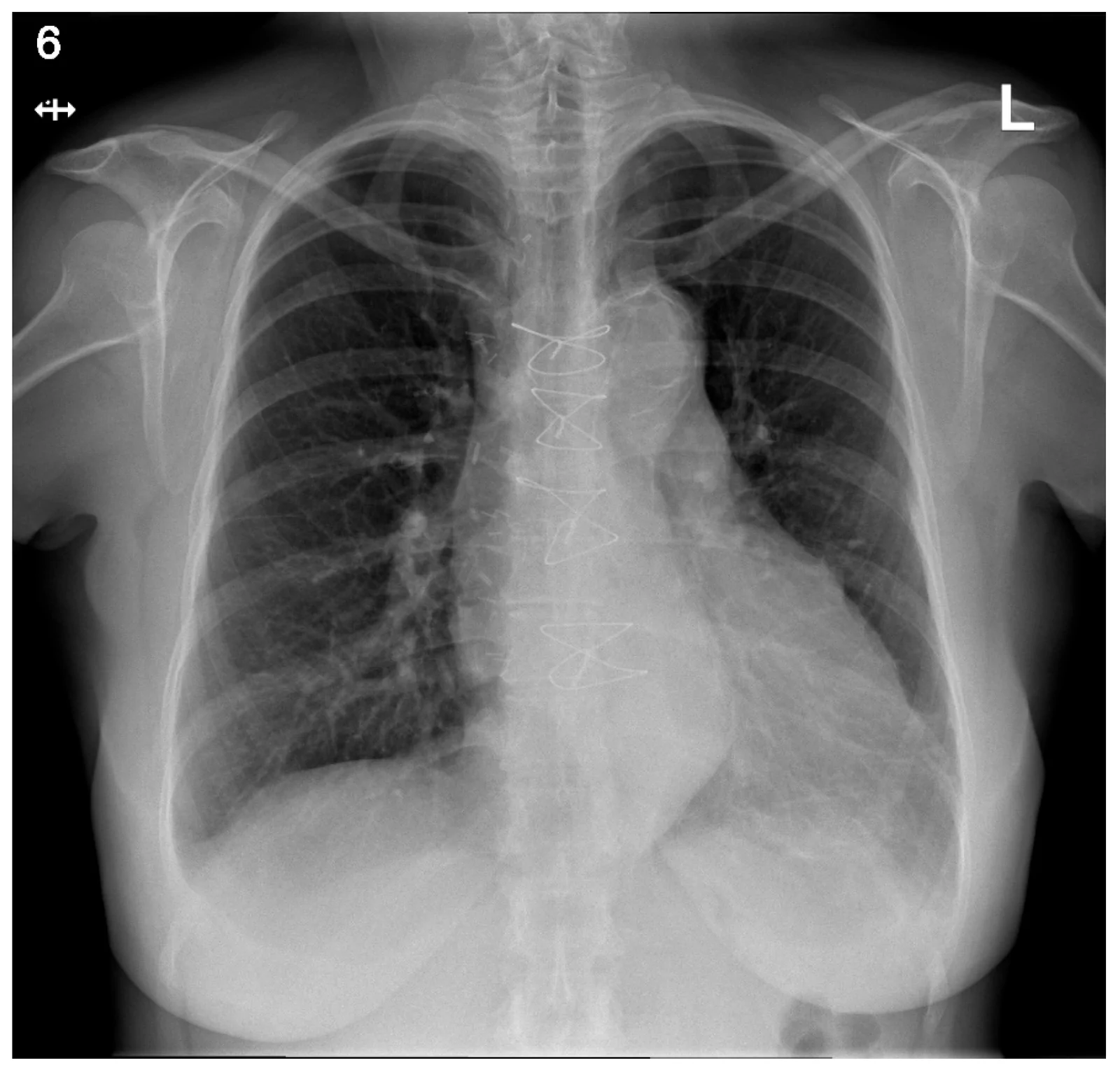
Chest X-ray showing normal heart size after transplantation.

The transplant procedure was successful, with immediate graft function and improved cardiac silhouette on chest X-ray (Figure 6). In the course of the first day PT, the patient’s condition deteriorated severely, manifesting with hemodynamic instability requiring pharmacological and pulmonary vasodilator support with adrenaline, norepinephrine, milrinone, and inhaled nitric oxide (iNO). The early postoperative course was further complicated by a severe coagulation disorder requiring multiple blood product transfusions guided by thromboelastography.

**Figure 7:**
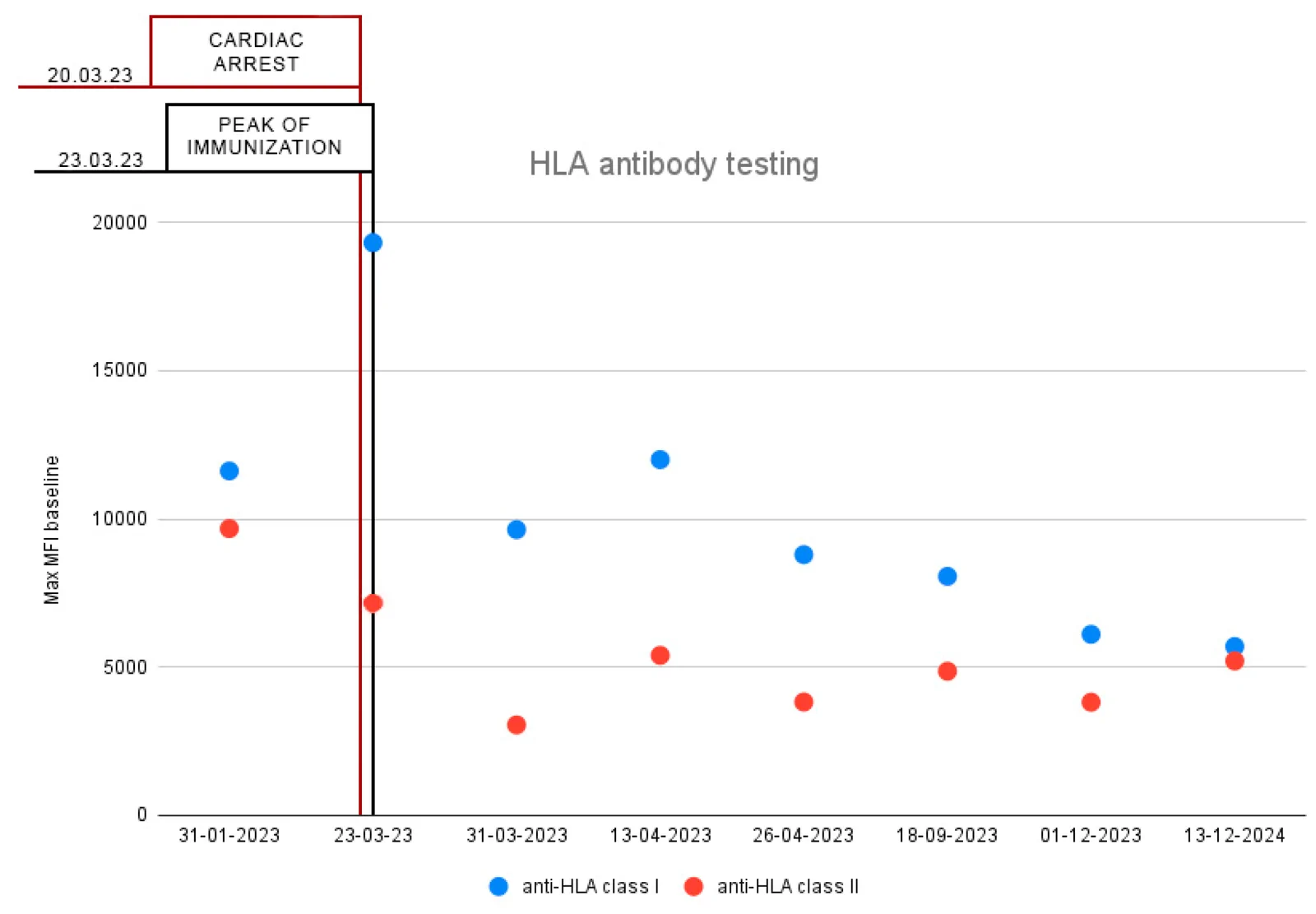
Time trends of changes in class I and II anti-HLA antibodies.

Afterwards, between 9 and 10 days PT, the patient developed severe bradycardia progressing to cardiac arrest lasting approximately 5 min. Advanced life support was initiated, resulting in the return of spontaneous circulation. Post-resuscitation echocardiography excluded cardiac tamponade but revealed myocardial stunning, particularly affecting the right ventricle. Hemodynamic stability was subsequently restored with continuous infusions of milrinone and norepinephrine. Later, slight clinical improvement was observed. Following the cardiac arrest, immunological testing continued to detect anti-HLA class I and class II antibodies; however, their donor specificity could not be confirmed. As illustrated in Figure 7, anti-HLA class I and class II antibody reactivity generally decreased during post-transplant follow-up, although fluctuations were observed. The highest recorded MFI values were observed on 23 March 2023, three days after the cardiac arrest. However, donor specificity could not be confirmed, and the available data do not establish a causal relationship between anti-HLA antibody reactivity and the cardiac arrest.

**Figure 8:**
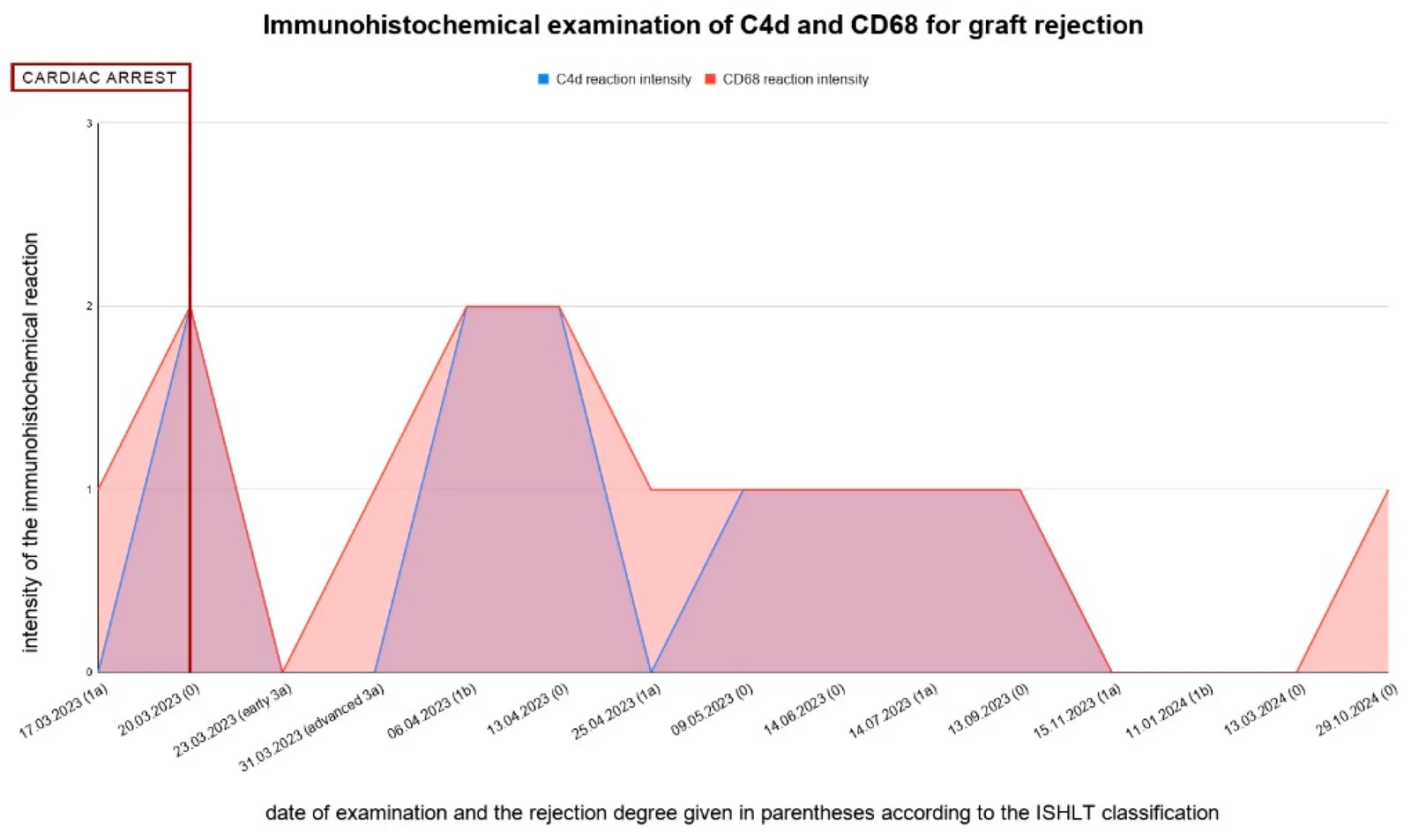
Semiquantitative assessment of C4d and CD68 immunohistochemical staining in endomyocardial biopsy specimens.

Additionally, immunohistochemical assessment of EMB specimens demonstrated C4d and CD68 staining, which was semiquantitatively scored on a scale of 0–2 (Figure 8). These findings were considered descriptive and interpreted alongside the biopsy-based diagnosis of acute cellular rejection (ACR) and the patient’s clinical course; they did not independently establish a definitive pathological antibody-mediated rejection classification.

On PT day 11, laboratory tests the day after cardiac arrest revealed elevated inflammatory markers and impaired renal function, with creatinine of 1.73 mg/dL, CRP of 93 mg/L, and NT-proBNP exceeding 70,000 pg/mL. Subsequently, CRP increased to 121.26 mg/L, accompanied by leukocytosis (15.32 × 10^9^/L). Antibiotic therapy was initiated, followed by improvement in inflammatory markers and initial clinical stabilization. On the 14th day PT, planned EMB confirmed cellular rejection (ISHLT grade 3A). From this point, steroid therapy was intensified. Unfortunately, NT-proBNP remained elevated and myocardial edema developed and was observed on echocardiography. At this point, the patient was transferred to the intensive care unit, where an additional course of plasma exchange, consisting of 5–7 daily sessions beyond the initial desensitization protocol, was considered in response to the clinical deterioration and biopsy findings. However, given the patient’s severe postoperative course, including respiratory failure requiring mechanical ventilation and worsening renal function requiring loop diuretic therapy, this additional course was not pursued.

Ultimately, the patient’s clinical condition gradually improved until she was discharged from the hospital 2 months after the transplant.

**Figure 9:**
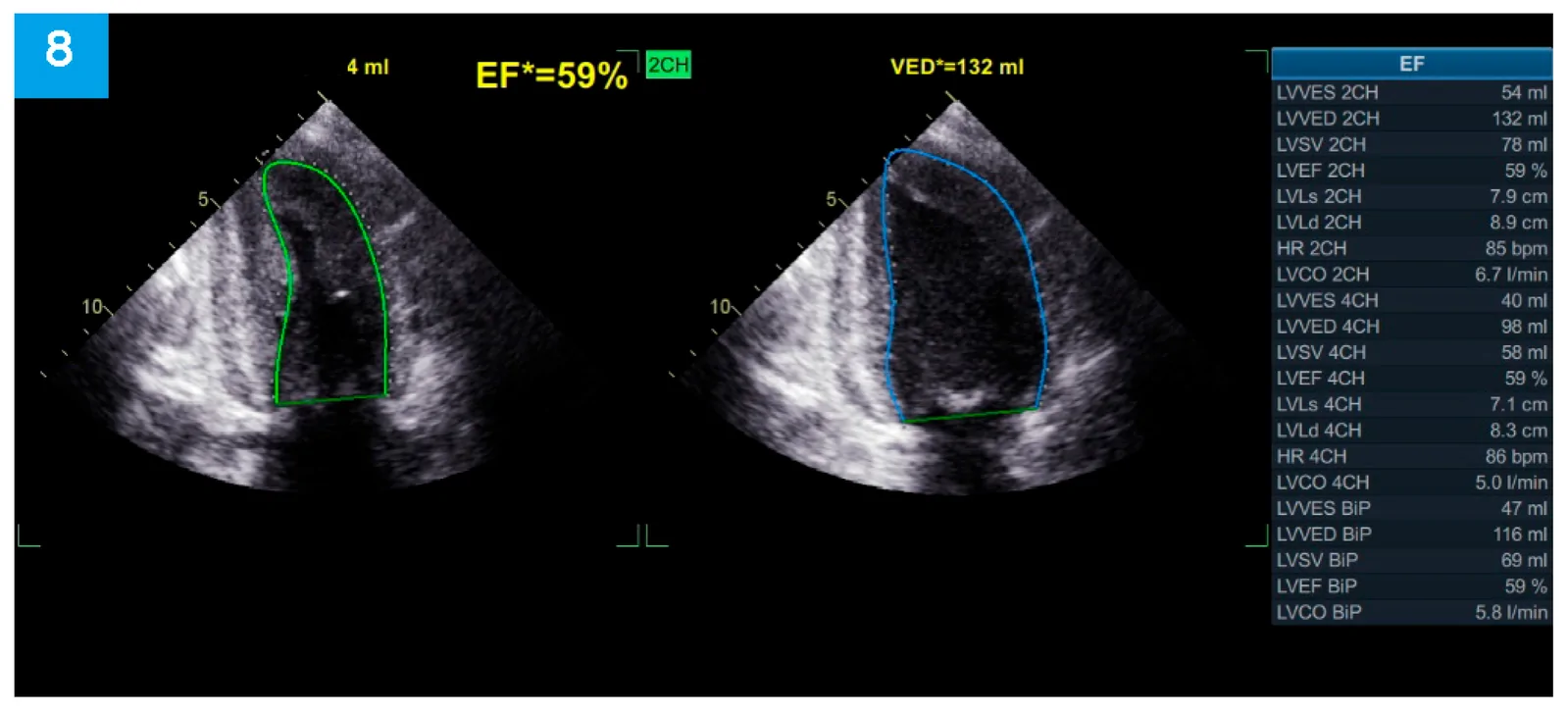
Echocardiographic parameters of the heart post-transplantation.

At 123 days post-transplant, during routine control, ACR was confirmed—ISHLT 1A. At that time the patient presented with NYHA class II. NT-proBNP remained around 1000 pg/mL. Tacrolimus dosing was adjusted to maintain a whole-blood trough concentration of 10–12 ng/ml for tacrolimus and 1.5–2.5 µg/mL for mycophenolic acid. At 185 days PT, the patient improved to NYHA class I. Despite persistent myocardial edema on echocardiography, ACR was again excluded (ISHLT grade 0). Left ventricular function remained preserved, with an ejection fraction of 59%, as shown in Figure 9, indicating overall graft stability despite earlier fluctuations in rejection severity. At 248 days PT, EMB revealed ISHLT Grade 1A rejection. By day 306 PT, the rejection grade changed to 1B, prompting an increase in tacrolimus dosage to maintain whole-blood trough concentrations within the target range, while mycophenolate mofetil therapy was unadjusted.

At 366 days PT, the patient experienced a temporary clinical deterioration to NYHA class III; however, this was not related to cardiovascular causes, but rather to vaginal bleeding and subsequent severe anemia of Hb 6.4 g% requiring transfusion. Despite this transient worsening, rejection was excluded by EMB (ISHLT Grade 0); after that, she was stabilized through adjustment of immunosuppressive therapy. This improvement allowed for a hysterectomy procedure to be carried out at 443 days PT to address underlying gynecological indications. Following the procedure, the patient reported clinical improvement to NYHA class II, with preserved left ventricular function and normalization of laboratory parameters. At 597 days PT another EMB showed no evidence of ACR (ISHLT grade 0), and she remained clinically stable, with normal blood count. The patient continues routine post-transplant follow-up, including clinical assessment, laboratory testing, and evaluation of graft function by echocardiography.

## 3. Discussion and Conclusions

HTx in patients with TA is exceptionally rare, and the limited available evidence provides little guidance regarding optimal candidate selection, immunological management and long-term outcomes [19,20,21]. Therefore, the findings of this case should be interpreted cautiously and require confirmation in larger clinical studies.

We emphasize that TA is characterized by persistent immune dysregulation, encompassing both cellular and humoral immune responses, including increased B-cell activity and the presence of autoantibodies [9,10]. However, pre-transplant anti-HLA alloimmunization typically requires prior exposure to alloantigens, for example, through blood transfusion, pregnancy, or prior transplant [15]. Therefore, in this particular case, TA may have constituted an immunologically active background, but its direct contribution to anti-HLA sensitization cannot be established [22,23,24].

A particularly noteworthy feature of this case was the temporal overlap between anti-HLA antibody positivity and a life-threatening episode of asystole. However, this association does not prove a causal relationship between antibody levels and cardiac arrest. Early after transplantation, EMB confirmed ACR (ISHLT grade 3A), while anti-HLA antibody testing provided complementary information regarding the patient’s humoral alloimmune status. This finding underscores the importance of integrating histopathological assessment with immunological monitoring in highly sensitized HTx recipients. Although previous studies have suggested potential effects of immune-mediated mechanisms on myocardial electrical stability and antibody-associated cardiac injury [25,26], further studies are needed to determine whether HLA sensitization is mechanistically associated with life-threatening arrhythmias after HTx.

Furthermore, standard PT immunosuppressive therapy may exert a dual therapeutic effect in patients with TA. In addition to preventing graft rejection, agents such as corticosteroids, calcineurin inhibitors, and biologic therapies (e.g., tocilizumab) may simultaneously suppress the activity of the underlying vasculitic process [27,28,29]. Although PT immunosuppression may theoretically influence TA activity, this possibility remains speculative because longitudinal vascular follow-up data were unavailable. The optimal immunosuppressive guidance in this specific population remains to be clearly defined, and further studies are needed to evaluate long-term outcomes [30].

Perioperative desensitization strategies based on the Hannover protocol described by Sommer et al. have been reported as an approach to managing pre-transplant sensitization in carefully selected HTx candidates [18,31]. In this case, the protocol required individualized modifications based on the patient’s clinical and immunological status. Specifically, due to hemodynamic instability, rituximab was omitted. Despite the modification and a complicated early posttransplant course, including severe graft dysfunction and biopsy-proven rejection, the patient ultimately achieved stable graft function during long-term follow-up. This case illustrates the challenges of implementing an individualized perioperative desensitization strategy in a highly sensitized recipient and highlights the need for close clinical and immunological monitoring. Its efficacy cannot be established from a single case [32,33]. This case also highlights the value of individualized, multidisciplinary care, reflecting recent advances in HF management.

In conclusion, this case describes the clinical course of a highly sensitized recipient who underwent HTx following an individualized perioperative desensitization strategy. The efficacy and broader applicability of the modified protocol cannot be established from a single case.

## 4. Limitations

Our primary objective in this publication was to present a clinically complex case that reached a successful outcome through the application of innovative solutions at the intersection of immunology and transplantology. However, this case presentation is subject to a number of limitations, stemming from its retrospective nature and the fact that it concerns only a single patient.

First, the available medical records did not contain sufficiently detailed information to reconstruct the initial diagnostic process for TA, assess disease activity prior to transplantation, track previous disease-specific treatments, or determine the extent of persistent vascular lesions. Consequently, it was not possible to establish how the immunosuppressive regimen administered after transplantation influenced TA activity.

Second, there was no crossmatch result available for the donor ultimately selected for transplantation, which limited the ability to assess immunological compatibility between the donor and the recipient. Although the presence of antibodies against HLA class I and II antigens was monitored (data available regarding their peak reactivity levels), a comprehensive retrospective analysis could not be performed due to the lack of detailed DSA profiles and quantitative measurements taken at the time of the cardiac arrest.

Third, the unavailability of detailed histopathological descriptions from endomyocardial biopsies precluded an independent verification of antibody-mediated rejection based on current ISHLT criteria. While immunohistochemical findings concerning the presence of C4d, CD68, and CD3 were recorded, a full interpretation was not possible in the absence of the corresponding histopathological descriptions. Consequently, the role of antibody-dependent mechanisms in the occurrence of cardiac arrest remains unclear.

Finally, it should be emphasized that, as this report concerns a single case, the clinical outcome does not allow for determination of the efficacy of the modified desensitization protocol employed, nor does it permit definitive conclusions regarding the feasibility of its broader application in other highly sensitized heart transplant recipients.

## Data Availability

All data generated or analyzed during this study are included in this published article. Additional data supporting the findings of this study are available from the corresponding author upon reasonable request.

## Abbreviations

ACR—acute cellular rejection; AR—aortic regurgitation; CDC-CM—complement-dependent cytotoxicity crossmatch; CRP—C-reactive protein; DSA—donor-specific antibodies; EF—ejection fraction; EMB—endomyocardial biopsy; HF—heart failure; HTx—heart transplantation; HLA—human leukocyte antigen; ISHLT—International Society for Heart and Lung Transplantation; MR—mitral regurgitation; NT-proBNP—N-terminal pro-B-type natriuretic peptide; NYHA—New York Heart Association; PRA—panel reactive antibodies; PT—post-transplant; TA—Takayasu arteritis; TTE—transthoracic echocardiography; TR—tricuspid regurgitation.
